# The presence of atypical mycobacteria in the mouthwashes of normal subjects: Role of tap water and oral hygiene

**DOI:** 10.4103/1817-1737.37890

**Published:** 2008

**Authors:** Siraj O. Wali, M. M. Abdelaziz, A. B. Krayem, Y. S. Samman, A. N. Shukairi, S. A. Mirdad, A. S. Albanna, H. J. Alghamdi, A. O. Osoba

**Affiliations:** Department of Medicine, King Khalid National Guard Hospital, Jeddah, Saudi Arabia; * *Department of Epidemiology, Medical College, Um Al-Qura University, Makkah, Saudi Arabia*; ** *Department of Microbiology, King Khalid National Guard Hospital, Jeddah, Saudi Arabia*

**Keywords:** Mycobacterial isolates, nontuberculous mycobacteria, oral flora, oral hygiene

## Abstract

**BACKGROUND:**

The nontuberculous mycobacteria (NTM) have been found in different environmental sources. They tend to colonize different body surfaces and secretions. The purpose of this study is to evaluate the presence of NTM in the oral cavity of healthy individuals and its relationship to tap water or oral hygiene.

**MATERIALS AND METHODS:**

One hundred sixty-seven healthy subjects were recruited. Three consecutive early morning mouthwashes using tap water were performed and examined for the presence of *Mycobacterium tuberculosis* (MTB) and NTM. In addition we obtained mouthwashes from 30 control healthy individuals with good oral hygiene using sterile water and examined these for the presence of MTB and NTM.

**RESULTS:**

NTM was isolated from the mouthwash of 44 (26.3%) subjects that used tap water. On the other hand, NTM was isolated from the mouthwash of 10 (33%) subjects that used sterile water. Age, gender, social class oral hygiene and the regular use of toothbrush made no statistically significant differences in the isolation rate of NTM.

**CONCLUSION:**

The rate of isolation of NTM from mouthwash is high in normal subjects. It is independent of oral hygiene, the use of tap water or teeth brushing. Smear-positive sputum could be NTM rather than *M. tuberculosis*. Tuberculosis polymerase chain reaction or culture confirmation is essential in developing countries to avoid the unnecessary use of antituberculosis therapy when the clinical suspicion is very low.

Nontuberculous mycobacteria (NTM) are ubiquitous microorganisms, present in soil, water, house dust, domestic animals, milk and plant materials.[1–4] They tend to contaminate clinical specimens or transiently colonize body surfaces and secretions.[5] In addition, NTM were also reported to contaminate artificial habitats such as tap water and sewage sludge. Person-to-person transmission does not occur and it seems that environmental exposure is important for NTM human infections.[6,7]

Although NTM is an important pathogen in patients with chronic lung disease, it can also colonize the respiratory tract of individuals with normal lungs.[5] The isolation of NTM in sputum alone is not equivalent to NTM disease according to the American Thoracic Society (ATS) diagnostic criteria for NTM infection.[4]

Studies performed in Saudi Arabia among symptomatic subjects with chronic lung disease have shown that two-thirds of the mycobacterium isolates from oral and respiratory specimens are NTM.[8–10] In addition, Nsanze *et al.*, observed that NTM was isolated from the oral cavity and urine specimens of 50% of healthy individuals in the southern parts of Saudi Arabia.[11] However, it is not known whether NTM may colonize the oral cavity of individuals with no lung disease. The purpose of this study is to evaluate the presence of NTM in the oral cavity of healthy Saudi individuals and its relationship to tap water or poor oral hygiene. The clinical implication of our findings is also discussed.

## Materials and Methods

Health care workers and subjects with no lung symptoms and disease attending our Ambulatory Outpatient Department (AOD) were recruited into the study. Only asymptomatic subjects with normal chest radiograph were included. Patients with clinical or radiological findings suggestive of active or old tuberculosis were excluded. All patients were above 18 years of age and they consented to their recruitment into the study. Data regarding their occupation and the use of toothbrush were collected. Oral hygiene was evaluated by internists.

All subjects were divided into three social classes based on their occupation: low, medium and high. Occupations of subjects were converted to social class according to the registrar general's classification (Class 1-6): Class 1 and 2, High; Class 3 and 4, Medium; and Class 5 and 6, Low.[12] Oral hygiene was assessed for the presence of caries, calculus and gingival inflammation. The presence of one of these findings indicated poor oral hygiene.

Three consecutive early morning mouthwashes using tap water were performed before toothbrush and examined for the presence of mycobacteria. Patients were asked to swish the water in their mouth early in the morning and before teeth brushing and gargle for 3 seconds before spitting it out in the container. Samples of tap water used for mouth washing were also tested in the same way.

In addition, mouth-washing samples from 30 individuals with good oral hygiene were also obtained using sterile water to make sure that the isolation of NTM in the first group was not due to NTM-contaminated water. Specimens from sterile water were evaluated for the presence of mycobacteria.

Specimens were decontaminated and concentrated. Direct smears from the deposit were performed using Ziel-Neelsen stain. The remaining deposit was cultured on Lowenstein Jensen's (LJ) medium and BacT / Alert TB liquid culture bottles (Organon Teknika; Durham N.C.). LJ cultures were incubated for 8 weeks and BacT / Alert TB liquid cultures were incubated for 14 days. Smears from positive cultures were also performed using Z-N stain for confirmation of AFB. Molecular assays were performed on all isolates for MTB DNA using Amplicor Polymerase Chain Reaction (PCR) MTB (Roche Diagnostic Systems, Branchburg, NJ) according to manufacturer's instructions. PCR MTB DNA positive isolates indicated *Mycobacterium tuberculosis*, while negative isolates were considered MTB.[13]

Speciation of mycobacterial isolates was carried out by the Multi-Gen Detection System (Genotype Mycobacteria) by Symbiosis (Via San Carlo, 10, 14023 Cocconata, Asti, Italy) according to manufacturer's instructions. Briefly, DNA of the mycobacteria was amplified with biotin-labeled primers. After denaturation, the DNA was hybridized with probes for the different mycobacteria species. A reading card was used for the interpretation of the various bands obtained. This reverse hybridization assay allows the identification of only 16 different species of mycobacteria.

## Statistical analysis

Chi-square test was used to compare proportions across the different groups (NTM-positive group versus NTM-negative group). Fisher's exact test was used when cell size was less than 5. For continuous variables, we used T-test for independent variables. Data analysis was carried out using SPSS software package, version 75 for windows.

## Results

One hundred sixty-seven subjects were studied. The frequency of low, middle and high social class was 58% (97 / 167), 25% (42 / 167) and 17% (28 / 167) respectively. NTM was isolated from the mouthwash of 44 subjects (26.3%) using tap water. There was no difference between NTM-positive group and NTM-negative group in gender and age distribution [Table 1]. However, the frequency of low social class was higher among non tuberculous mycopacteria-positive group compared with that among non tuberculous mycopacteria-negative group [Table 1]. Additionally, mouth-washing samples using sterile water were obtained from 30 subjects of middle social class (mean age 39.2 ± 16.8; 20 females and 10 males) and used as control. In this control group, NTM was isolated from 33% of specimens (10 out of 30 samples), indicating that the presence of NTM in the mouth was independent of tap water, at least partly.

**Table 1:** Nontuberculous mycobacteria isolation in relation to demographics and social classes

Demographics	NTM positive (44/167, 26.3%)	NTM negative (123/167, 73.7%)	*P*-value
Age	40.6 (+14.1)	44.9 (+15.7)	0.09
Female	25 (56.8%)	66 (53.6%)	0.72
Male	19 (43.2%)	57 (53.6%)	0.72
Low social class	31 (70.5%)	66 (53.7%)	0.05
Middle social class	10 (22.7%)	32 (26.0%)	
High social class	3 (6.8%)	25 (20.3%)	0.05

NTM - Nontuberculous mycobacteria

All subjects of high social class had satisfactory oral hygiene (100%) compared to medium (59.5%) and low social class individuals (16.7%) (*P* = 0.005). Similarly, most of the subjects in the high social class were regular users of toothbrush (96.4%) compared to medium (69.0%) and low social class subjects (39.2%) (*P* = 0.005). However, the use of toothbrush and the status of oral hygiene were not different between NTM-PG and NTM-NG [Table 2].

**Table 2:** Nontuberculous mycobacteria isolates from mouthwashes of 167 subjects and relationship to oral hygiene

Level of oral hygiene	NTM positive (44/167, 26.3%)	NTM negative (123/167, 73.7%)	*P*-value
Poor oral hygiene	30 (68)	78 (63)	0.13
Regular use of toothbrush	28 (62.6)	66 (53.7)	0.33

NTM - Nontuberculous mycobacteria

Mycobacterium species analysis was performed in 29 specimens randomly selected from the subjects that used tap water. Mixed mycobacterial isolates were identified in three samples. Fourteen species could not be identified. *Mycobacterium gordonae* was the most common species. *M. fortuitum*, *M. kansasii* and *Mycobacterium avium complex* (MAC) were isolated from 3, 2 and 2 subjects respectively [Table 3].

**Table 3:** Speciation of 29 mycobaterial isolates from 44 positive mouthwashes

Mycobacterium species	Number (%)
*Mycobacterium gordonae*	8 (27.6)
*Mycobacterium kansasii*	2 (6.9)
*Mycobacterium fortuitum*	3 (10.3)
*Mycobacterium avium complex*	2 (6.9)
Unidentified species	14 (48.3)

Although tap water samples used for mouthwashes were taken as control and examined for MTB and NTM, NTM species was detected in only five specimens of them. *M. gordonae* was isolated from two samples while *M. fortuitum*, *M. kansasii* and unidentified mycobacterium species were isolated from the remaining three samples. Similarly, samples of sterile water used were taken as control, but none revealed NTM species.

## Discussion

Our study demonstrated that NTM can present in the oral cavity in asymptomatic healthy individuals, irrespective of the oral hygiene and use of toothbrush. Although there were many epidemiological studies of NTM worldwide using skin testing or clinical specimens such as sputum culture, NTM are not considered part of normal flora of the oral cavity. Our findings suggest that these organisms may contaminate normal mouth flora in some individuals in our environment.

Most species of NTM are normal inhabitants of the environment and are found in water, soil and aerosol. Unlike MTB, there is no evidence of person-to-person spread of NTM.[14,15] Also, these environmental mycobacteria are not normally regarded as part of the normal mouth flora. Therefore, in an attempt to explain their occurrence in the mouthwash of normal individuals, We examined the samples of tap water used for the mouthwashes and it was found that NTM were present in only 5 samples, 2 of them used by subjects with positive mouthwash for NTM. This may suggest that the presence of NTM in these two individuals was due to contamination from tap water. In a study of contamination of clinical specimens, Arnow *et al.,* found that cultures from tap water, ice and iced drinking water yielded *M. gordonae* in significant numbers.[16] Furthermore, culture survey showed mycobacterial contamination of 8 of 34 (24%) sputum samples collected immediately after a tap water mouth rinse.[16] Other studies have also shown that NTM could be recovered from natural drinking water, including Mycobacterium avium complex (MAC), *M. kansasii, M. malmoense* and *M. fortuitum*.[15,17] In contrast to natural water, NTM could not be recovered from bottled water, suggesting that these organisms are likely to be found in ground water.[18] These findings suggest that contaminated water may be the source of NTM in sputum samples or mouthwash. Therefore, the presence of these species in the mouthwashes of some of our subjects may be attributed partly to mycobacteria-contaminated tap water, though this would not offer a full explanation of our findings. The detection of mycobacteria in only 5 samples of tap water suggests that other factors may be involved. Furthermore, examination of the samples of mouthwashes using sterile water revealed NTM in 33% of the cases (10 out of 30 samples), indicating that the presence of NTM in the mouth was independent of the use of tap water.

Although it is tempting to postulate that the ingestion of mycobacteria-contaminated foodstuff, such as meat, vegetable, dairy product and eggs, may play an important role in NTM being part of the oral flora, this seems to be an unlikely possibility as the presence of NTM in these foodstuff is rare.[19–20] Yajko and co-workers studied the presence of MAC in 397 food samples and isolated mycobacterium species in only 2 samples.[19] In contrast, raw milk samples examination was shown to yield significant numbers of NTM species.[3,20] However, its role in the transmission of NTM needs further evaluation. This may be of particular importance in Saudi Arabia, where drinking raw milk is a common practice in some areas of the country and presently plays an important role in transmission of brucellosis.[21] In addition, soil samples have yielded a wide variety of these NTM and studies have demonstrated the presence of NTM in house dust.[6,7] However, further studies are needed to determine whether raw milk, soils or house dust have a role in explaining our findings of the presence of NTM in mouthwash of healthy individuals.

Our study has shown that there was a trend to isolate NTM with increasing frequency from individuals from the low social class rather than from the high social class. Although poor oral hygiene was significantly more frequent among individuals from the low social class compared to those from the higher class oral hygiene status does not appear to influence the frequency of NTM isolation. Similarly, the rate of regular use of toothbrush was found to be higher in the high social class group when compared with the low social class group but again had no effect on the frequency of NTM isolation. These findings suggest that the high prevalence of NTM in the mouthwashes from the low social class subjects was independent of oral hygiene, regular use of toothbrush and different life style. However, we did not examine the technique of tooth brushing and the use of Miswak (an alternative to tooth brushing in Saudi Arabia), which might have influenced our findings.

*Mycobacterium gordonae* was the most frequently identifiable species in our study, being isolated from eight samples. *Mycobacterium fortitium* was isolated from three specimens, while *M. kansasii* and MAC were isolated from two samples each. Although these species may be isolated from clinical specimens from individuals with no clinical disease, they can also be implicated in pulmonary diseases, particularly in immunocompromized patients. The clinical implications and the health hazards of the presence of NTM in a high percentage of individuals in our study as part of the mouth flora needs to be examined further. In patients with clinical and radiological findings suggestive of tuberculosis, the presence of acid-fast bacilli in the sputum may not necessarily indicate that the patient is suffering from infection due to *M. tuberculosis*; hence the possibility of NTM-induced disease should be considered. This is of particular importance in immunocompromized patients.

Our findings have many clinical implications, especially in developing countries, where tuberculosis is highly endemic.[22,23] In many developing countries, because of lack of resources, the diagnosis and treatment initiation of pulmonary tuberculosis is usually based on smear-positive sputum samples prior to culture or PCR confirmation. However, in view of the results of this study, this practice may lead to improper and overuse of antituberculous medications, especially in those with low clinical suspicion of tuberculosis. Therefore, in such circumstances emphasis should be placed on appropriate confirmatory tests such as culture and / or PCR, where available, to confirm the diagnosis.

Our study has some limitations, which should be addressed. There was some concern that the tap water used for mouth wash was contaminated with NTM from the start and therefore the yield of NTM was not from the oral cavity of the subject tested. However, using sterile water in the second group showed that the presence of NTM was independent of tap water used (at least, partly). Additionally, individuals in the second group (used sterile water) had good oral hygiene, while subjects in the first group had good and poor oral hygiene. However, if we stratify the first group into subjects with good and poor oral hygiene, we will find that 59 persons had good oral hygiene and 23% (14 / 59) of them were positive for NTM, which is comparable with the second group using sterile water. Another limitation of the study was that mycobacterium species analysis was performed in 29 specimens randomly selected from subjects that used tap water. This was mainly because of lack of funding; at the time, we had only 29 kits available. Finally, the authors acknowledge that clinical implication of this study may be not applicable in developed countries and in areas where PCR and culture were carried out routinely.

## Conclusion

Our study indicates that NTM may contaminate the mouthwashes of normal subjects, but unrelated to oral hygiene or teeth brushing. The water supply could partly explain this result but does not explain the entire findings. Therefore, other factors such as ingestion of raw milk, soil and aerosols need to be studied. The importance of sputum culture or PCR assay confirmation of Z-N positive sputum is emphasized, especially in developing countries where these tests are not carried out routinely.
